# Efficacy and safety of maintenance therapy with anlotinib for advanced cholangiocarcinoma after first-line chemotherapy and the variations in efficacy based on different neutrophil-to-lymphocyte ratio (NLR)

**DOI:** 10.1186/s12957-024-03472-9

**Published:** 2024-07-29

**Authors:** Hui Li, Jue Zhang, Lili Gu

**Affiliations:** 1 Department of Clinical Oncology, Affiliated Nanjing Tianyinshan Hospital, Pharmaceutical University, Nanjing, 210000 China; 2 Department of Clinical Oncology, Affiliated Jinling Hospital, Medical School of Nanjing University, Nanjing, 210000 China; 3 Department of General surgery, Affiliated Jinling Hospital, Medical School of Nanjing University, No. 305 Zhongshan East Road, Xuanwu District, Nanjing City, 210000 Jiangsu Province China

**Keywords:** Cholangiocarcinoma, Anlotinib, Neutrophil-to-lymphocyte ratio, Clinical efficacy

## Abstract

**Objective:**

This study aimed to evaluate the clinical efficacy and safety of anlotinib as maintenance therapy in patients with advanced cholangiocarcinoma following first-line chemotherapy.

**Methods:**

This retrospective study enrolled 154 patients with advanced biliary tract cancer admitted to the hospital between January 2020 and December 2022. All patients received first-line intravenous chemotherapy with gemcitabine combined with cisplatin, oxaliplatin, or tegafur. Among the 106 patients who achieved disease control, 47 received oral anlotinib hydrochloride (12 mg daily, 2 weeks on/1 week off) as maintenance therapy. Clinical efficacy, including ORR, DCR, DOR, PFS, and OS, was compared between the anlotinib maintenance and non-maintenance groups. Subgroup analysis based on NLR levels was also performed.

**Results:**

Among the 47 anlotinib maintenance patients, the ORR was 21.28% and the DCR was 51.06%. The median DOR was 36 weeks, and the median PFS was 43 weeks in the anlotinib group, versus 28 weeks and 38 weeks in the non-maintenance group, respectively. The median OS was not reached in the anlotinib group but was 48 weeks in the non-maintenance group. Patients receiving anlotinib maintenance had significantly longer DOR, PFS, and OS (all *p* < 0.05). Patients with low NLR levels had better survival benefits from anlotinib.

**Conclusion:**

Maintenance therapy with anlotinib demonstrates potential efficacy and a reliable safety profile in patients with advanced cholangiocarcinoma following first-line treatment. The efficacy of anlotinib therapy appears to be influenced by NLR levels. Further validation with larger sample sizes is warranted to strengthen the robustness and reliability of the results.

## Introduction

Cholangiocarcinoma, the second most prevalent primary hepatic malignancy following hepatocellular carcinoma, is a malignant tumor originating from the epithelium of the bile ducts [1]. Its incidence and mortality rates have been rapidly increasing worldwide in recent years. While surgical resection is the primary curative treatment for early-stage cholangiocarcinoma, a significant number of patients are diagnosed with unresectable or metastatic disease at the time of diagnosis, thereby missing the opportunity for potentially curative surgery [2, 3]. Apart from patients with specific target mutations, chemotherapy remains the primary treatment for biliary tract cancer. However, the short duration of response (DOR) and the high incidence of complications after first-line progression pose challenges for patients who require second-line treatment. Currently, second-line treatment mainly consists of oxaliplatin-based regimens, which have a low efficacy rate and result in a modest increase in overall survival (OS) from 5.3 months to only 6.2 months compared to a placebo. Therefore, there is a need to explore alternative treatment modalities for patients who respond well to first-line therapy, and this has become an area of active research [4]. Maintenance therapy with less intensive chemotherapy has shown promise in improving survival outcomes for patients with advanced cholangiocarcinoma, but a standardized approach has yet to be established [5].

Anlotinib, a multi-targeted receptor tyrosine kinase inhibitor (TKI), exerts antitumor effects on various solid tumors primarily by inhibiting key pathways involved in tumor growth and angiogenesis, including the vascular endothelial growth factor receptor (VEGFR), fibroblast growth factor receptor (FGFR), and platelet-derived growth factor receptor (PDGFR) pathways [6, 7]. By targeting angiogenesis and cell proliferation signaling pathways, anlotinib demonstrates efficacy in inhibiting tumor growth [8]. Clinical trials evaluating anlotinib in non-small cell lung cancer, hepatocellular carcinoma, renal carcinoma, gastric carcinoma, and soft tissue sarcoma have reported favorable clinical activity [9, 10]. Furthermore, preclinical and clinical investigations have identified several other promising anti-angiogenic TKIs, such as regorafenib, lenvatinib, and apatinib, which have demonstrated antitumor activity in animal models and patients with cholangiocarcinoma [11, 12]. High blood pressure is the most common adverse drug reaction associated with anti-tumor angiogenesis drugs. Anlotinib, as a vascular endothelial growth factor receptor (VEGFR) inhibitor, can suppress the activity of VEGFR. However, vascular endothelial growth factor (VEGF) itself induces the release of nitric oxide, which promotes vasodilation. When the VEGF pathway is inhibited, the concentration of nitric oxide decreases, resulting in increased blood pressure. Additionally, the decrease in microvessel density caused by anlotinib may lead to increased peripheral vascular resistance, contributing to hypertension [13]. The exact mechanism of hand-foot syndrome induced by anlotinib is not yet clear. It may be related to the expression of tumor necrosis factor-alpha and the blockade of downstream signaling pathways after inhibition of VEGFR [13, 14]. Proteinuria caused by anlotinib is primarily attributed to the important role of VEGF in maintaining the integrity of glomerular endothelium. Disruption of endothelial cells and podocytes occurs when the VEGF pathway is inhibited, affecting the function of the glomerular filtration barrier [14, 15]. Anlotinib, as a small molecule multitarget tyrosine kinase inhibitor, may cause fatigue due to disturbances in endocrine regulation, anemia, and mineral metabolism disorders [16]. Mucositis caused by molecular targeted drugs is most commonly observed in the oral cavity. The mechanism underlying oral mucositis is mainly attributed to the generation of free radicals by the drug, resulting in epithelial damage, activation of inflammatory factors leading to tissue and mucosal cell injury, and colonization of oral flora accompanied by inflammatory cell infiltration [17].

Given that a significant proportion of unresectable intrahepatic cholangiocarcinoma (ICC) cases are advanced, anlotinib—being a novel therapeutic option—may potentially improve treatment outcomes by prolonging progression-free survival and overall survival.

In light of these considerations, we conducted an open-label, single-arm, single-center clinical study to evaluate the clinical efficacy of anlotinib maintenance therapy in patients with advanced cholangiocarcinoma following first-line chemotherapy, as well as its impact on serum markers. The primary objective of this study was to investigate whether anlotinib maintenance therapy following chemotherapy can improve the survival of patients with advanced cholangiocarcinoma. The results of this study are expected to provide valuable insights for larger-scale clinical trials in this patient population and contribute to the advancement of treatment strategies for advanced cholangiocarcinoma. While the focus of this study is on intrahepatic cholangiocarcinoma (ICC), the maintenance therapy with anlotinib may have potential benefits and broad implications for the overall survival prognosis of other types of cancer. These findings suggest that anlotinib maintenance therapy could have a wide-ranging impact beyond ICC and may offer favorable outcomes in various malignancies.

## Patients and methods

### Study design and patient

A retrospective analysis was conducted on a cohort of 154 patients with advanced bile duct cancer who received treatment at our hospital. Among these patients, 106 achieved disease control after 12–18 weeks of first-line treatment. Among the patients who achieved disease control, 47 received maintenance therapy with anlotinib, while the remaining 59 patients received observation, all patients were evaluated every 6 weeks.

Prior to enrollment, all patients were provided with a detailed explanation of the study protocol and potential risks, and written informed consent was obtained from each participant. The study protocol was approved by the Ethics Committee of our hospital, and all procedures were conducted in accordance with the ethical principles outlined in the Declaration of Helsinki.

### Inclusion and exclusion criteria

The inclusion criteria were as follows: (1) age between 18 and 75 years, regardless of gender; (2) histologically confirmed diagnosis of advanced cholangiocarcinoma; (3) Eastern Cooperative Oncology Group (ECOG) performance status score of 0–2; (4) medical records and follow-up data are complete; (5) presence of at least one measurable lesion that can be accurately assessed according to the Response Evaluation Criteria in Solid Tumors (RECIST) version 1.1 [13]; and (6) achieving either a partial response (PR) or stable disease (SD) after 12–18 weeks of a gemcitabine-based first-line regimen.

The exclusion criteria were as follows: (1) previous treatment with anilotinib; (2) active or concurrent malignancies within the past five years; (3) significant organ dysfunction or poor physical condition; and (4) disease progression.

### Treatment regimen

First-line chemotherapy consisted of intravenous drip administration of gemcitabine (800mg/m^2^) primarily on days 1 and 8, combined with cisplatin (in the majority of cases), oxaliplatin, or tegafur gimeracil oteracil potassium capsule(S1). This chemotherapy regimen was repeated every 3 weeks. Patients who achieved a complete response (CR), partial response (PR) or stable disease (SD) received a maximum of 6 cycles of chemotherapy. Following the completion of chemotherapy, oral maintenance therapy with anlotinib was initiated after a two-week interval.

Maintenance therapy involved the administration of oral anlotinib hydrochloride at a dose of 12 mg once daily for all patients. Each dose consisted of one tablet, and the medication was taken continuously for two weeks, followed by a one-week drug holiday. Each cycle of maintenance therapy lasted for 21 days. The administration of anlotinib was suspended in case of disease progression (PD) or if the patient experienced intolerable adverse reactions. Depending on the severity of drug-related toxic reactions and the anticipated benefits, the dose could be reduced to 10 mg or 8 mg daily if the patient could not tolerate the 12 mg daily dose. However, if the patient could not tolerate the 8 mg daily dose, the treatment would be discontinued. It is important to note that once the dose is reduced, it cannot be increased back to the previous level.

### Observational endpoints

According to RECIST 1.1 criteria, treatment response was evaluated by an experienced radiologist who assessed tumor assessments every 6 weeks until disease progression. The efficacy endpoints included complete response(CR), partial response (PR), stable disease (SD), progressive disease (PD), disease control rate (DCR) on 24 weeks after the end of the last intravenous chemotherapy; progression-free survival (PFS), overall survival (OS), and duration of response (DOR). The DCR was calculated as the combination of the proportions of patients with complete response, partial response and stable disease. PFS was defined as the time from the start of chemotherapy to tumor recurrence or progression. OS was defined as the time from the start of chemotherapy to death, and DOR was measured as the time from the first assessment of disease control to the first assessment of disease progression. The safety of the treatment was assessed using the Common Terminology Criteria for Adverse Events (CTCAE version 5.0).

The neutrophil-to-lymphocyte ratio (NLR) was calculated based on the absolute neutrophil count and absolute lymphocyte count obtained from the most recent complete blood count within the 10 days prior to the initiation of anlotinib treatment. The study compared the differences in overall survival (OS) among patients with different NLR expression levels.

### Statistical analysis

Categorical data were presented as frequencies and percentages, while continuous data were expressed as mean ± standard deviation (SD). Survival curves were generated using the Kaplan-Meier method, providing median times and 95% confidence intervals (CIs) and log-rank test was adopted to report the P value. Statistical analysis was performed using SPSS version 23.0, and figures in the manuscript were generated using R software. Two-tailed tests were employed for all statistical tests, and a significance level of *P* < 0.05 was considered statistically significant.

## Results

### Baseline characteristics

A total of 47 patients with advanced cholangiocarcinoma, who demonstrated no PD following first-line chemotherapy, were included in this study. Table 1 presents the clinical baseline characteristics of the enrolled patients. Among the cohort, 29 cases (63.38%) were male, while 18 cases (36.17%) were female. The median age was 54 years, with an age range of 42 to 73 years. The ECOG performance status scores were distributed as follows: 0 in 16 cases (34.04%), 1 in 29 cases (61.70%), and 2 in 2 cases (4.26%). In terms of primary tumor location, 20 cases (42.55%) were classified as intrahepatic cholangiocarcinoma (ICC), 17 cases (36.17%) as extrahepatic cholangiocarcinoma (ECC), and 10 cases (21.28%) as gallbladder carcinoma (GBC). The metastatic status of the patients was categorized as follows: 8 cases (17.02%) with no distant metastasis but not amenable to surgical resection. 21 cases (44.68%) with one site of distant metastasis, 14 cases (29.79%) with two sites of metastasis, and 4 cases (8.51%) with more than three sites of metastasis. Regarding the response to first-line treatment, 12 cases achieved a PR, while 35 cases exhibited SD. Prior history of radiotherapy was reported in 5 cases (10.64%), while 42 cases (89.36%) had not undergone radiotherapy.

**Table 1:** Baseline profiles of participants

Characteristic	Patients	Percentage (%)
Age, years	54 (42–73)	
≤ 65 years old	30	63.83
>65 years old	17	36.17
Sex		
Female	29	61.70
Male	18	38.30
ECOG performance status		
0	16	34.04
1	29	61.70
2	2	4.26
Primary site		
ICC	20	42.55
ECC	17	36.17
GBC	10	21.28
Number of metastatic sites		
0	8	17.02
1	21	44.68
2	14	29.79
≥ 3	4	8.51
Best response to first-line chemotherapy		
PR	12	25.53
SD	35	74.47
Previous radiotherapy		
Yes	5	10.64
No	42	89.36
NLR		
≥ 2.54	30	63.83
<2.54	17	36.17

Note: ICC = Intrahepatic Cholangiocarcinoma, ECC = Extrahepatic Cholangiocarcinoma, GBC = Gall-Bladder Cancer, PR = Partial Response, SD = Stable Disease, NLR = Neutrophil-To-Lymphocyte Ratio

### Clinical efficacy analysis

Follow up until the end of August 2023, with no loss to follow-up. At the end of the follow-up period, a total of 47 patients were available for efficacy assessment, and the results are summarized in Table 2. At the end of the last intravenous chemotherapy for 24 weeks, among the 47 patients, none achieved CR, while 10 patients maintained PR with a reduction in maximum tumor diameter of more than 30%. Additionally, 14 patients maintained SD, while 23 patients experienced disease progression, including 18 deaths. Among the patients included in the study, the overall disease control rate (DCR) was found to be 51.06%. In patients with a neutrophil-to-lymphocyte ratio (NLR) of ≥ 2.54, there were 6 cases of partial response (PR), 8 cases of stable disease (SD), and 16 cases of progressive disease (PD), resulting in a DCR of 46.67% (14 out of 30 patients). In patients with an NLR of < 2.54, there were 4 cases of PR, 6 cases of SD, and 7 cases of PD, with a DCR of 58.83% (10 out of 17 patients). However, there was no significant difference observed in the objective response rate (ORR) and DCR between patients with an NLR of ≥ 2.54 and those with an NLR of < 2.54.

**Table 2:** Overall efficacy

Characteristic	total	NLR ≥ 2.54	NLR<2.54
N	47	30	17
PR	10	6	4
SD	14	8	6
PD	23	16	7
DCR	24	14	10

Note: NLR = Neutrophil-To-Lymphocyte Ratio, PR = Partial response, SD = Stable Disease, PD = Progressive Disease, DCR = Disease Control Rate

### Survival curve analysis

**Fig. 1:**
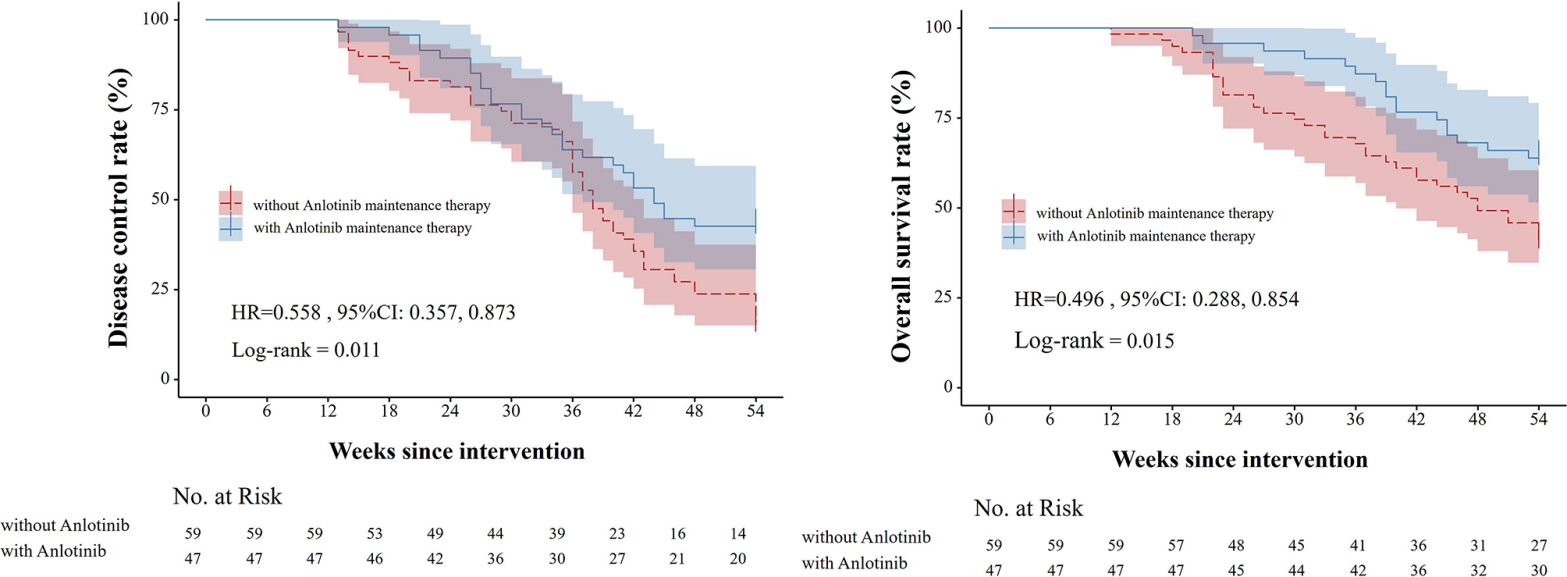
Difference in OS and PFS with Anlotinib Maintenance Therapy

**Fig. 2:**
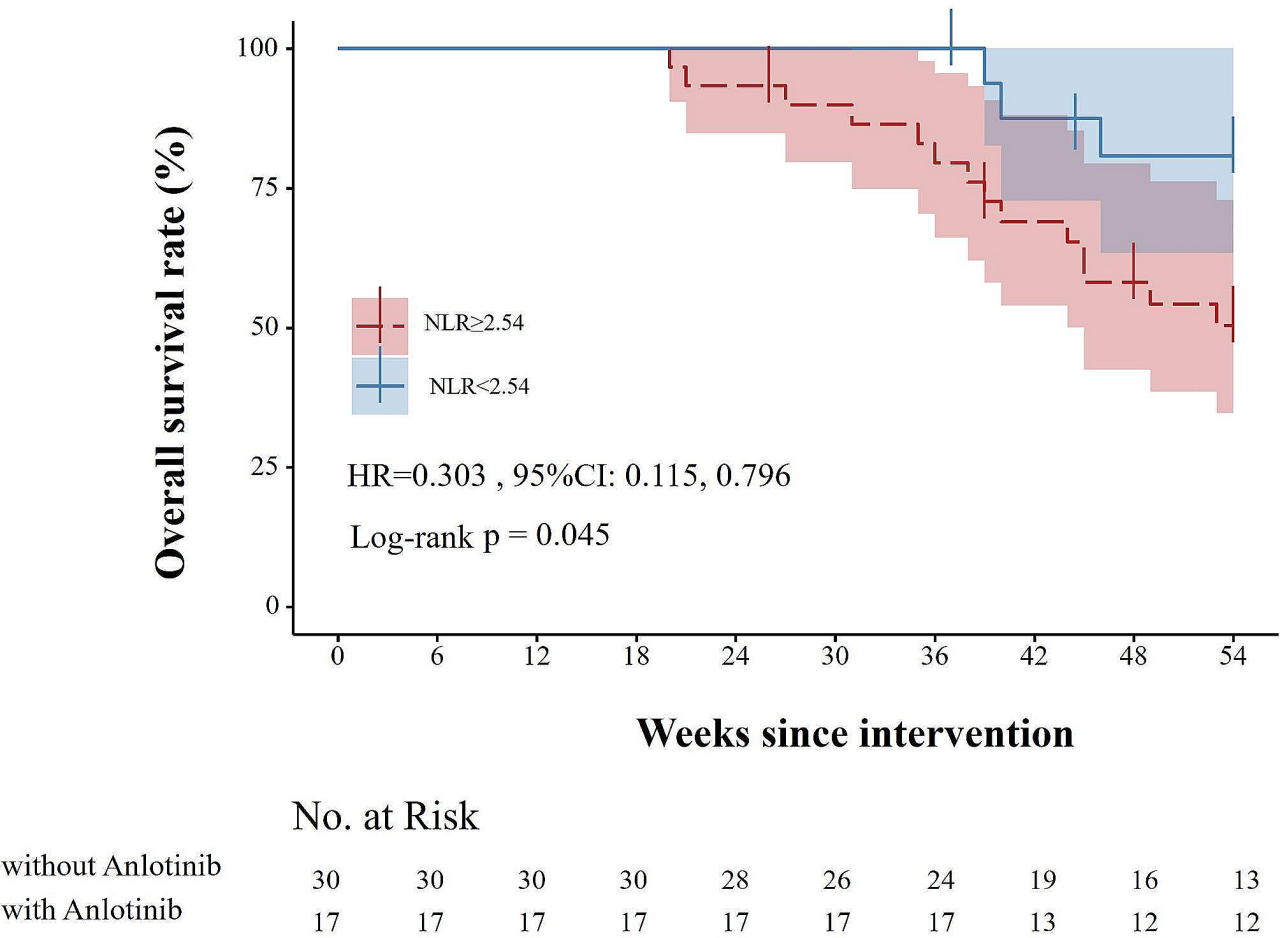
Differences in Overall Survival (OS) at Different NLR Levels

The study utilized the Kaplan-Meier method to analyze the differences in overall survival (OS) and progression-free survival (PFS) between patients who received anlotinib maintenance therapy and those who did not, as depicted in Fig. 1. Patients who underwent anlotinib maintenance therapy achieved a median duration of response (DOR) of 36 weeks, a median PFS of 43 weeks, with a follow-up period that was relatively short and did not reach the median OS. In contrast, among patients who did not receive anlotinib maintenance therapy, the median DOR was 28 weeks, a median PFS of 38 weeks, and the median OS was 48 weeks. Significantly longer DOR, PFS and OS were observed in patients who underwent anlotinib maintenance therapy (all *P* < 0.05).The Kaplan-Meier method was utilized to generate the overall survival curve, specifically for patients undergoing anlotinib maintenance therapy. These patients were further divided into different subgroups based on their Neutrophil-to-Lymphocyte Ratio (NLR). The impact of NLR on survival outcomes was assessed using the Kaplan-Meier method, as depicted in Fig. 2. In the study population, the median overall survival (OS) was not reached in either NLR subgroup. Patients with low NLR levels demonstrated better survival outcomes compared to those with high NLR levels (HR = 0.0303, 95% CI: 0.115, 0.796). This indicates that low NLR levels were associated with improved survival benefits.

### Adverse event analysis

Among the 47 patients, a total of 37 individuals experienced adverse events (AEs), including 31 cases of grade 1–2 AEs and 6 cases of grade 3–4 AEs. No grade 5 AEs or drug-related deaths were reported. The most commonly observed AEs included hand-foot syndrome, hypertension, fatigue, proteinuria, hematuria, lethargy, nausea and vomiting, anorexia, constipation, and elevated alanine transaminase (ALT) and aspartate transaminase (AST) levels, with a higher incidence of grade 1–2 severity. Notably, all these symptoms showed significant improvement with symptomatic treatment. Among patients experiencing grade 3–4 AEs, four cases required discontinuation of treatment due to intolerance. The incidence of adverse events is detailed in Table 3.

**Table 3:** Incidence of adverse events

Adverse events	All patients	Grade 1–2	Grade 3–4
HFS reaction	22	20	2
Fatigue	6	6	0
Proteinuria	12	11	1
Urine occult blood	16	16	0
hypertension	24	20	4
Vomiting and Diarrhea	10	10	0
Anorexia	14	13	1
Constipation	15	13	2
ALT/AST elevation	15	14	1

Note: HFS = Hand-foot symptom

## Discussion

Cholangiocarcinoma, the second most prevalent primary liver tumor, remains a formidable malignancy with a high mortality rate for the majority of patients [14]. The diagnosis often occurs at advanced stages, characterized by the dissemination of tumors to distant sites, rendering surgical resection less feasible [15]. Surgical resection is only a viable option for a fraction of cholangiocarcinoma patients, ranging from 30 to 40%, and even with adjuvant capecitabine treatment, the median overall survival remains a modest 53 months [16]. The current standard first-line treatment for cholangiocarcinoma involves a combination chemotherapy regimen of gemcitabine and cisplatin. While this therapeutic approach exhibits efficacy in certain patients, the majority of cholangiocarcinoma patients fail to attain satisfactory therapeutic outcomes [17]. A study conducted on patients with bile duct cancer revealed discouraging results in terms of treatment outcomes. The combination chemotherapy regimen of gemcitabine and cisplatin yielded a median progression-free survival (PFS) of only 2.8 months, with an overall survival (OS) of merely 11.7 months [18]. Moreover, the efficacy of second-line treatment for advanced bile duct cancer is generally low, and patients in subsequent lines of therapy often encounter high rates of tumor-related jaundice and biliary infections, which can hinder further treatment opportunities. Given the limited benefits and safety concerns associated with chemotherapy in bile duct cancer patients, there has been a shift in research focus towards molecular targeted therapy and immunotherapy strategies. Despite the administration of effective first-line treatment, there remains a risk of recurrence in bile duct cancer. In order to reduce the recurrence rate, some studies have suggested the feasibility of maintenance therapy. Maintenance therapy with molecular targeted drugs has emerged as a new option for patients with bile duct cancer following first-line chemotherapy [19].

In this study, the use of anlotinib as maintenance therapy following chemotherapy demonstrated several advantages over conventional first-line chemotherapy in patients with bile duct cancer. It resulted in a prolonged duration of response (DOR), progression-free survival (PFS), and overall survival (OS). Furthermore, the manageable adverse reactions associated with anlotinib make it a more clinically acceptable option for patients. Future investigations may involve exploring multi-drug combination therapies as first-line treatments or sequential treatment modalities to further extend DOR, PFS, and OS, with the ultimate goal of improving the quality of life for patients. Another domestic study suggested that combining anlotinib and sintilimab with gemcitabine and cisplatin chemotherapy might provide additional benefits for patients with advanced bile duct cancer [20]. Data on the combination of anlotinib with PD-1 inhibitors in second-line treatment for bile duct cancer, as well as the effectiveness of a four-drug combination comprising anlotinib, lenvatinib, PD-1 inhibitors, and chemotherapy as a first-line treatment, have shown promising results and have become a hot topic in the treatment of advanced bile duct cancer [21]. Findings from a phase Ib clinical study have also indicated favorable efficacy of anlotinib in combination therapy for patients with advanced bile duct cancer [22].

Anlotinib, an independently developed tyrosine kinase inhibitor in China, exerts potent anti-angiogenic and antitumor effects by targeting vascular endothelial growth factor receptor and fibroblast growth factor receptor [23]. Comparative studies have indicated that anlotinib possesses superior antiangiogenic activity compared to sunitinib and sorafenib [24]. Furthermore, maintenance therapy utilizing anlotinib has demonstrated promise in reducing recurrence in patients afflicted with unresectable or metastatic advanced soft tissue sarcoma, osteosarcoma, and smooth muscle tumors that have shown positive responses to chemotherapy [25, 26]. Importantly, anlotinib treatment has been shown to induce apoptosis and mesenchymal-epithelial transition, significantly inhibiting tumor growth in patient-derived xenograft models derived from cholangiocarcinoma patients [27]. Moreover, in cholangiocarcinoma cell lines, anlotinib exhibited a substantially lower IC50 compared to other reported tyrosine kinase inhibitors [28]. Additionally, the combination of anlotinib and gemcitabine has proven effective in suppressing the growth of cholangiocarcinoma cell lines while concurrently increasing the expression of cleaved PARP/PARP and cleaved caspase-3/caspase-3 proteins [29].

Furthermore, our study explored the relationship between different NLR levels and clinical efficacy. Notably, no significant differences were found in ORR and DCR among varying NLR levels. However, low NLR levels were associated with superior survival outcomes when compared to high NLR levels. NLR serves as an established peripheral blood inflammatory indicator and a biomarker for predicting the prognosis of various tumor types. Numerous investigations have demonstrated that high NLR levels are indicative of poorer clinical prognoses [30, 31]. A study focusing on distal resectable cholangiocarcinoma observed that elevated preoperative NLR levels were predictive of inferior long-term prognoses, which aligns with the findings of our study [32]. A meta-analysis further confirmed that elevated pretreatment NLR levels were independently associated with shorter OS, PFS, and poorer clinicopathologic features in patients with advanced cholangiocarcinoma [33].

This retrospective study has limitations, including selection bias leading to a non-representative sample. To mitigate selection bias, strict inclusion and exclusion criteria were implemented to ensure that the study sample was as representative as possible of the target population. To minimize recall bias, treatment response was assessed by the same experienced radiologist based on RECIST 1.1 criteria. Tumor evaluation was performed every 6 weeks until disease progression. By considering these limitations and employing appropriate methods to reduce their impact, the results of retrospective studies can be more balanced in their evaluation.

Implementing anlotinib maintenance therapy in a clinical setting may face several challenges. Toxicity management is a significant concern as anlotinib, being a targeted therapy, can induce a range of adverse reactions such as hypertension, hand-foot syndrome, and gastrointestinal reactions. Close monitoring of patients’ toxicities and timely implementation of appropriate management strategies are necessary to mitigate the impact of these adverse reactions on patients’ quality of life. Anlotinib, as a novel targeted therapy, may come with a higher treatment cost, which can impose a burden on both patients and healthcare institutions. Cost-effectiveness analysis is essential while implementing anlotinib maintenance therapy to assess its actual contribution to prolonged patient survival and improved quality of life, as well as its cost-effectiveness compared to other treatment options. Although anlotinib has shown promising efficacy and tolerability in some clinical trials, larger-scale randomized controlled trials are still needed to further validate its effectiveness and safety in maintenance therapy. These trials can help determine the optimal application strategies of anlotinib in different cancer types and treatment stages, providing more robust scientific evidence for its widespread use in clinical practice.

This study evaluated the real-world application and impact of anlotinib in a clinical setting, assessing its treatment effectiveness and influence on patients’ quality of life. The next steps required to translate the study results into clinical practice include a comprehensive assessment of the treatment’s efficacy as demonstrated by the research. This includes determining the impact of anlotinib maintenance therapy on patient survival, disease progression, and quality of life, as well as its advantages and disadvantages compared to standard treatment or alternative therapeutic approaches. Defining the indications for anlotinib use is crucial, clearly defining the indications for anlotinib maintenance therapy, including factors such as patient disease type, disease stage, genotype, and other relevant considerations. This helps ensure the appropriate use of anlotinib and maximizes patient benefits. Detailed treatment protocols and guidelines need to be established before introducing anlotinib into clinical practice. This includes determining the dosage, administration schedule, treatment duration, toxicity management strategies, etc., to ensure that patients can receive the treatment safely and effectively. Prior to the implementation of anlotinib in clinical practice, relevant education and training should be provided to clinical physicians and healthcare teams. This helps ensure that healthcare professionals have knowledge about the indications, precautions, toxicity reactions, and management of anlotinib, thereby enhancing the quality and safety of treatment. By considering the aforementioned measures and closely collaborating with healthcare teams in clinical practice, the translation of research results into clinical practice can be effectively achieved, providing patients with more effective treatment options.

## Conclusion

In conclusion, maintenance therapy utilizing anlotinib shows promise as an effective treatment for patients with advanced cholangiocarcinoma following first-line treatment. The combination of targeted agents with chemotherapy, such as the use of anlotinib and sintilimab, as well as the incorporation of anti-angiogenic drugs like regorafenib and lenvatinib, holds potential for improving outcomes in this challenging disease. Furthermore, NLR levels may serve as a prognostic indicator in advanced cholangiocarcinoma, although larger studies are needed to validate these findings. Ongoing research and clinical trials are crucial to further explore and optimize the use of molecularly targeted therapies and immunotherapeutic strategies in the management of cholangiocarcinoma, with the ultimate goal of improving patient outcomes and survival rates.

## Data Availability

No datasets were generated or analysed during the current study.

## References

[1] Razumilava N, Gores GJ. Cholangiocarcinoma Lancet. 2014;383:2168–79. 24581682 10.1016/S0140-6736(13)61903-0 PMC4069226

[2] Rizvi S, Gores GJ. Pathogenesis, diagnosis, and management of cholangiocarcinoma. Gastroenterology. 2013;145:1215–29. 24140396 10.1053/j.gastro.2013.10.013 PMC3862291

[3] Blechacz B, Komuta M, Roskams T, Gores GJ. Clinical diagnosis and staging of cholangiocarcinoma. Nat Rev Gastroenterol Hepatol. 2011;8:512–22. 21808282 10.1038/nrgastro.2011.131 PMC3331791

[4] Elvevi A, Laffusa A, Scaravaglio M, Rossi RE, Longarini R, Stagno AM, Cristoferi L, Ciaccio A, Cortinovis DL, Invernizzi P, Massironi S. Clinical treatment of cholangiocarcinoma: an updated comprehensive review. Ann Hepatol. 2022;27:100737. 35809836 10.1016/j.aohep.2022.100737

[5] Rizvi S, Khan SA, Hallemeier CL, Kelley RK, Gores GJ. Cholangiocarcinoma - evolving concepts and therapeutic strategies. Nat Rev Clin Oncol. 2018;15:95–111. 28994423 10.1038/nrclinonc.2017.157 PMC5819599

[6] Shen G, Zheng F, Ren D, Du F, Dong Q, Wang Z, Zhao F, Ahmad R, Zhao J. Anlotinib: a novel multi-targeting tyrosine kinase inhibitor in clinical development. J Hematol Oncol. 2018;11:120. 30231931 10.1186/s13045-018-0664-7 PMC6146601

[7] Zhong Q, Liu Z. Efficacy and safety of Anlotinib in patients with Advanced Non-small Cell Lung Cancer: a real-world study. Cancer Manag Res. 2021;13:4115–28. 34045898 10.2147/CMAR.S304838 PMC8149213

[8] Syed YY, Anlotinib. First Global Approval Drugs. 2018;78:1057–62. 29943374 10.1007/s40265-018-0939-x

[9] Lei T, Xu T, Zhang N, Zou X, Kong Z, Wei C, Wang Z. Anlotinib combined with osimertinib reverses acquired osimertinib resistance in NSCLC by targeting the c-MET/MYC/AXL axis. Pharmacol Res. 2023;188:106668. 36681369 10.1016/j.phrs.2023.106668

[10] Ye H, Li Z, Liu K, Zhang F, Cheng Z. Anlotinib, a novel TKI, as a third-line or further-line treatment in patients with advanced non-small cell lung cancer in China: a systemic review and meta-analysis of its efficacy and safety. Med (Baltim). 2021;100:e25709. 10.1097/MD.0000000000025709 PMC8202555 34114981

[11] Huang C, Wen Q, Chen J, Zhong H, Xiang X, Xiong J, Deng J. FDFT1/FGFR2 rearrangement: a newly identified anlotinib-sensitive FGFR2 variant in cholangiocarcinoma. Cancer Treat Res Commun. 2022;31:100568. 35477128 10.1016/j.ctarc.2022.100568

[12] Saborowski A, Vogel A, Segatto O. Combination therapies for targeting FGFR2 fusions in cholangiocarcinoma. Trends Cancer. 2022;8:83–6. 34840108 10.1016/j.trecan.2021.11.001

[13] Schwartz LH, Litière S, de Vries E, Ford R, Gwyther S, Mandrekar S, Shankar L, Bogaerts J, Chen A, Dancey J, Hayes W, Hodi FS, Hoekstra OS, Huang EP, Lin N, Liu Y, Therasse P. Wolchok JD and Seymour L. RECIST 1.1-Update and clarification: from the RECIST committee. Eur J Cancer. 2016;62:132–7. 27189322 10.1016/j.ejca.2016.03.081 PMC5737828

[14] Kendall T, Verheij J, Gaudio E, Evert M, Guido M, Goeppert B, Carpino G. Anatomical, histomorphological and molecular classification of cholangiocarcinoma. Liver Int. 2019;39(Suppl 1):7–18. 30882996 10.1111/liv.14093

[15] Vithayathil M, Khan SA. Current epidemiology of cholangiocarcinoma in western countries. J Hepatol. 2022;77:1690–8. 35977611 10.1016/j.jhep.2022.07.022

[16] Zhang H, Yang T, Wu M, Shen F. Intrahepatic cholangiocarcinoma: Epidemiology, risk factors, diagnosis and surgical management. Cancer Lett. 2016;379:198–205. 26409434 10.1016/j.canlet.2015.09.008

[17] Sarkis Y, Al Soueidy A, Kourie HR. Will advanced cholangiocarcinoma become a targetable malignancy? Crit Rev Oncol Hematol. 2021;159:103233. 33482346 10.1016/j.critrevonc.2021.103233

[18] Okusaka T. Cholangiocarcinoma: is it time for a revolution? Expert Rev Gastroenterol Hepatol. 2021;15:467–70. 33840344 10.1080/17474124.2021.1915766

[19] Cho SY, Hwang H, Kim YH, Yoo BC, Han N, Kong SY, Baek MJ, Kim KH, Lee MR, Park JG, Han SS, Lee WJ, Park C, Park JB, Kim JY, Park SJ, Woo SM. Refining classification of Cholangiocarcinoma Subtypes via Proteogenomic Integration reveals new therapeutic prospects. Gastroenterology. 2023;164:1293–309. 36898552 10.1053/j.gastro.2023.02.045

[20] Zhou J, Sun Y, Zhang W, Yuan J, Peng Z, Wang W, Gong J, Yang L, Cao Y, Zhao H, Chen C, Wang W, Shen L, Zhou A. Phase ib study of anlotinib combined with TQB2450 in pretreated advanced biliary tract cancer and biomarker analysis. Hepatology. 2023;77:65–76. 35491432 10.1002/hep.32548 PMC9970018

[21] Guo X, Shen W. Latest evidence on immunotherapy for cholangiocarcinoma. Oncol Lett. 2020;20:381. 33154779 10.3892/ol.2020.12244 PMC7608025

[22] Mahipal A, Kommalapati A, Tella SH, Lim A, Kim R. Novel targeted treatment options for advanced cholangiocarcinoma. Expert Opin Investig Drugs. 2018;27:709–20. 30124336 10.1080/13543784.2018.1512581

[23] Rimini M, Casadei-Gardini A. Angiogenesis in biliary tract cancer: targeting and therapeutic potential. Expert Opin Investig Drugs. 2021;30:411–8. 33491502 10.1080/13543784.2021.1881479

[24] Deng Z, Liao W, Wei W, Zhong G, He C, Zhang H, Liu Q, Xu X, Liang J, Liu Z. Anlotinib as a promising inhibitor on tumor growth of oral squamous cell carcinoma through cell apoptosis and mitotic catastrophe. Cancer Cell Int. 2021;21:37. 33422069 10.1186/s12935-020-01721-x PMC7796634

[25] Li S, Anlotinib. A Novel targeted drug for bone and soft tissue Sarcoma. Front Oncol. 2021;11:664853. 34094958 10.3389/fonc.2021.664853 PMC8173120

[26] Cui Q, Mao Y, Hu Y, Ma D, Liu H. Anlotinib in recurrent or metastatic endometrial cancer. Int J Gynecol Cancer 2022. 10.1136/ijgc-2022-003345 35606048

[27] Song F, Hu B, Cheng JW, Sun YF, Zhou KQ, Wang PX, Guo W, Zhou J, Fan J, Chen Z, Yang XR. Anlotinib suppresses tumor progression via blocking the VEGFR2/PI3K/AKT cascade in intrahepatic cholangiocarcinoma. Cell Death Dis. 2020;11:573. 32709873 10.1038/s41419-020-02749-7 PMC7381674

[28] Fraveto A, Cardinale V, Bragazzi MC, Giuliante F, De Rose AM, Grazi GL, Napoletano C, Semeraro R, Lustri AM, Costantini D, Nevi L, Di Matteo S, Renzi A, Carpino G, Gaudio E, Alvaro D. Sensitivity of human intrahepatic cholangiocarcinoma subtypes to Chemotherapeutics and Molecular targeted agents: a study on primary cell cultures. PLoS ONE. 2015;10:e0142124. 26571380 10.1371/journal.pone.0142124 PMC4646673

[29] Fan S, Ge Y, Liu J, Liu H, Yan R, Gao T, Fan X, Xiao Z, An G. Combination of anlotinib and gemcitabine promotes the G0/G1 cell cycle arrest and apoptosis of intrahepatic cholangiocarcinoma in vitro. J Clin Lab Anal. 2021;35:e23986. 34462984 10.1002/jcla.23986 PMC8529129

[30] Lino-Silva LS, Salcedo-Hernández RA, García-Pérez L, Meneses-García A, Zepeda-Najar C. Basal neutrophil-to-lymphocyte ratio is associated with overall survival in melanoma. Melanoma Res. 2017;27:140–4. 28125448 10.1097/CMR.0000000000000333

[31] Nakaya A, Kurata T, Yoshioka H, Takeyasu Y, Niki M, Kibata K, Satsutani N, Ogata M, Miyara T, Nomura S. Neutrophil-to-lymphocyte ratio as an early marker of outcomes in patients with advanced non-small-cell lung cancer treated with nivolumab. Int J Clin Oncol. 2018;23:634–40. 29442281 10.1007/s10147-018-1250-2 PMC6097082

[32] Chen Q, Yang LX, Li XD, Yin D, Shi SM, Chen EB, Yu L, Zhou ZJ, Zhou SL, Shi YH, Fan J, Zhou J, Dai Z. The elevated preoperative neutrophil-to-lymphocyte ratio predicts poor prognosis in intrahepatic cholangiocarcinoma patients undergoing hepatectomy. Tumour Biol. 2015;36:5283–9. 25672606 10.1007/s13277-015-3188-6

[33] Tang H, Lu W, Li B, Li C, Xu Y, Dong J. Prognostic significance of neutrophil-to-lymphocyte ratio in biliary tract cancers: a systematic review and meta-analysis. Oncotarget. 2017;8:36857–68. 28415734 10.18632/oncotarget.16143 PMC5482704

